# Novel Insight into Metabolism Mechanism of Biogenic Amines During Fermentation of Chinese Traditional Fermented Mandarin Fish (*Chouguiyu*) Based on Metabolism Pathway and Correlation Network

**DOI:** 10.3390/foods14162863

**Published:** 2025-08-18

**Authors:** Jun Li, Daqiao Yang, Yongqiang Zhao, Di Wang, Hui Huang, Chunsheng Li

**Affiliations:** 1 Guangdong Provincial Key Laboratory of Lingnan Specialty Food Science and Technology, Key Laboratory of Green Processing and Intelligent Manufacturing of Lingnan Specialty Food, Ministry of Agriculture and Rural, College of Light Industry and Food, Zhongkai University of Agriculture and Engineering, Guangzhou 510225, China; lijun@zhku.edu.cn; 2 Key Laboratory of Aquatic Product Processing, Ministry of Agriculture and Rural Affairs, National R&D Center for Aquatic Product Processing, South China Sea Fisheries Research Institute, Chinese Academy of Fishery Sciences, Guangzhou 510300, China; zhaoyq@scsfri.ac.cn (Y.Z.); wangdi@scsfri.ac.cn (D.W.); huanghuigd@aliyun.com (H.H.); 3 College of Life Sciences, Linyi University, Linyi 276005, China

**Keywords:** fermented mandarin fish, biogenic amine, synthesis, degradation, metagenome, correlation network, food safety

## Abstract

A complex microbial community gives the possibility to produce biogenic amines in traditional fermented foods. In this study, the metabolism mechanisms of biogenic amines during fermentation of fermented mandarin fish *Chouguiyu* were revealed based on the metabolic pathways and correlation analysis. Functional genes based on KEGG orthology related to biogenic amine metabolism were selected from the metagenome and were used to construct the biogenic amine metabolic pathways in *Chouguiyu*. A total of 91 and 75 genera were related to the synthesis and degradation of biogenic amines, respectively. High concentrations of cadaverine and putrescine were observed, while the other biogenic amines were detected in relatively low concentrations. The metabolic mechanisms of various biogenic amines were illuminated by correlation network maps between biogenic amines and microbial synthesis/degradation enzymes. *Lactococcus*, *Flavobacterium*, *Tessaracoccus*, and *Yoonia* could only degrade and not produce biogenic amines. *Acinetobacter* and *Enterococcus* possessed more abundant enzymes for degradation than synthesis. Amine oxidase (K00276), diamine N-acetyltransferase (K00657), and gamma-glutamylputrescine synthase (K09470) were the main biogenic amine degradation enzymes in the microbial community. *Lactococcus garvieae*, *Flavobacterium gelidilacus*, *Tessaracoccus antarcticus*, *Yoonia vestfoldensis*, *Acinetobacter haemolyticus*, and *Enterococcus ureasiticus* were the main microbial species for biogenic amine degradation and could be isolated as the potential strains for biogenic amine degradation in fermented foods.

## 1. Introduction

Biogenic amines (BAs) in foods are mainly produced by the decarboxylation of amino acids under the action of microbial decarboxylases [1]. Excessive BAs can cause a variety of harmful reactions, such as headaches, rashes, nausea, diarrhea, flushing, and neurotoxicity [2]. BAs are easily produced in fermented foods because of the high concentrations of free amino acids and complex microbial community [3]. Many researchers have studied the BA synthesis and degradation ability of microorganisms in fermented foods after isolation and identification [4,5]. However, it is difficult to give a comprehensive understanding of the microbial metabolism mechanism of BAs in fermented foods.

With the development of microbial analysis, high-throughput sequencing technology can comprehensively analyze the complex microbial community in fermented foods [6,7]. Many studies have discovered BA-producing and BA-degrading microorganisms through correlation analysis between microbial communities and BAs [8,9]. However, correlation analysis only considers whether the abundance of the microbial community is consistent with the change in BA content and ignores the relevant enzymes for BA metabolism in these microorganisms. It cannot determine the core microorganisms that contribute to the synthesis and degradation of BAs in fermented foods. Metagenomics can predict microbial function by analysis of the whole genetic material of microbial community [10,11]. It provides an effective method to select the enzymes related to BA metabolism from microorganisms in fermented foods [12].

*Chouguiyu* is a traditional fermented food made from mandarin fish in China, and it has a unique flavor and taste. Most studies have focused on the study of changes in free amino acids [13], volatile flavor compounds [14], and microbial community [11,15,16] during fermentation of *Chouguiyu*. There are plenty of microorganisms in *Chouguiyu* due to the low-salt fermentation process, which can not only generate unique flavor and taste substances but also produce BAs. However, there is a lack of research on the changes in BAs during fermentation of *Chouguiyu*. Moreover, the synthesis and degradation mechanisms of BAs based on microbial metabolism have rarely been reported.

Therefore, in this study, metagenome sequencing was performed using an Illumina Hiseq Xten to analyze the whole genetic material of the microbial community during *Chouguiyu* fermentation. The key genes that were associated with the synthesis and degradation of various BAs in the microbial community were selected after KEGG orthology (KO) annotation. Combined with the results of BA concentrations, the metabolism mechanisms of different BAs during fermentation of *Chouguiyu* were revealed based on the BA metabolic pathways and correlation networks between microorganisms and BAs. The core microorganisms for BA degradation in *Chouguiyu* were also identified. This study is expected to provide a comprehensive understanding of the synthesis and degradation mechanisms of BAs in *Chouguiyu*, which is helpful in the isolation of functional strains for the degradation of BAs.

## 2. Materials and Methods

### 2.1. Fermentation of Chouguiyu

*Chouguiyu* was made by fermenting mandarin fish coming from Guangdong, China, (*Siniperca chuatsi*, av. 500 g, frozen and thawed before fermentation) at 15–20 °C for 8 days in Anhui, China, according to previous studies [11,15,16]. Briefly, frozen mandarin fish was thawed, gutted, and cleaned. Then, the prepared fish was soaked in brine (6% salt and 0.02% spices). Fermentation used wild microbiota (no starter cultures). Three samples of *Chouguiyu* were collected on Day 1 (D1), Day 2 (D2), Day 4 (D4), Day 6 (D6), and Day 8 (D8) for further study.

### 2.2. Metagenome Sequencing of Microbial Community in Chouguiyu

The microorganisms in *Chouguiyu* were obtained after centrifugation of fermented liquid in each sample. Shotgun metagenomics was adopted to analyze the microbial community, as per previous studies [11,15,16]. Briefly, total genomic DNA was extracted from microorganisms using the E.Z.N.A.^®^ Soil DNA Kit (Omega Bio-tek, Norcross, GA, USA). After the verification of DNA purity, the genomic DNA from microorganisms was fragmented to construct the DNA library using NEXTFLEX^®^ Rapid DNA-Seq (Bioo Scientific, Austin, TX, USA). The DNA library was then used for paired-end sequencing through the Illumina Hiseq Xten (Illumina Inc., San Diego, CA, USA).

### 2.3. Gene Assembly and Functional Annotation

Sequencing data were assembled using MEGAHIT (version 1.1.2) to obtain the contigs. Open reading frames (ORFs) from each assembled contig were predicted using MetaGene. CD-HIT (version 4.6.1) was used to cluster the gene sequences (90% identity and 90% coverage). The longest gene in each class was selected as the representative sequence to construct the non-redundant gene set. The obtained genes were aligned to the KEGG and COG databases to obtain their annotation. The BA metabolic pathways in the microbial community of *Chouguiyu* were constructed based on the KO in the KEGG pathways. The abundance of enzymes from microbial genera was calculated based on the sum of KO abundance related to BA metabolism. Representative sequences of non-redundant gene catalogs were aligned to the NCBI NR database with an e-value cutoff of 1e-5 using Diamond (version 0.8.35).

### 2.4. Determination of Biogenic Amines

The quantification of tryptamine, β-phenylethylamine, putrescine, cadaverine, histamine, tyramine, spermidine, and spermine was carried out as previously described [17] with some modifications. Briefly, a total of 5.00 g of samples was added to centrifuge tubes with 10 mL perchloric acid (0.4 mol/L) and was ground for 20 min at 40 Hz using a Multi-Tube Vortexer (EFAA-HM-01, Yalin, Shanghai, China). After centrifugation at 4000 r/min for 20 min using a high-speed tabletop refrigerated centrifuge (TDZ5-WS, Xiangyi, Changsha, Hunan, China), the supernatant (1 mL) was mixed with 100 μL sodium hydroxide (2 mol/L) and 300 μL saturated sodium bicarbonate. The derivative reaction was performed by adding dansyl chloride (1 mL) into the mixture, followed by incubation in the dark at 40 °C for 45 min. The BAs were detected using a high-performance liquid chromatograph (LC-20AD, Shimadzu, Nakagyo-ku, Kyoto, Japan) equipped with an ultraviolet detector (SPD-M20A, Shimadzu, Nakagyo-ku, Kyoto, Japan) on the WondaCract ODS-2 column (Shimadzu-GL, Nakagyo-ku, Kyoto, Japan). Solvent A and solvent B were acetonitrile and ammonium acetate, respectively. Elution program was performed as follows: 0.1 min, 55% B; 7 min, 65% B; 14 min, 70% B; 20 min, 70% B; 27 min, 90% B; 30 min, 100% B; 31 min, 100% B, 32 min, 55% B, and 37 min, 55% B with a flow rate of 0.80 mL/min. The BA contents of each sample were calculated according to the standard curves (Table S1).

### 2.5. Statistical Analysis

The statistical analysis was conducted using SPSS 20.0 software (SPSS Inc., Chicago, IL, USA) with one-way analysis of variance and multiple comparison Tukey tests. The correlation network maps between microbial BA metabolism enzymes and BA contents were built according to Pearson’s correlation by Cytoscape v3.9.1. Heatmaps were produced using Excel (Microsoft Office Home and Student 2019, Redmond, WA, USA).

## 3. Results and Discussion

### 3.1. Metagenomic Analysis of Microbial Community in Chouguiyu

**Figure 1:**
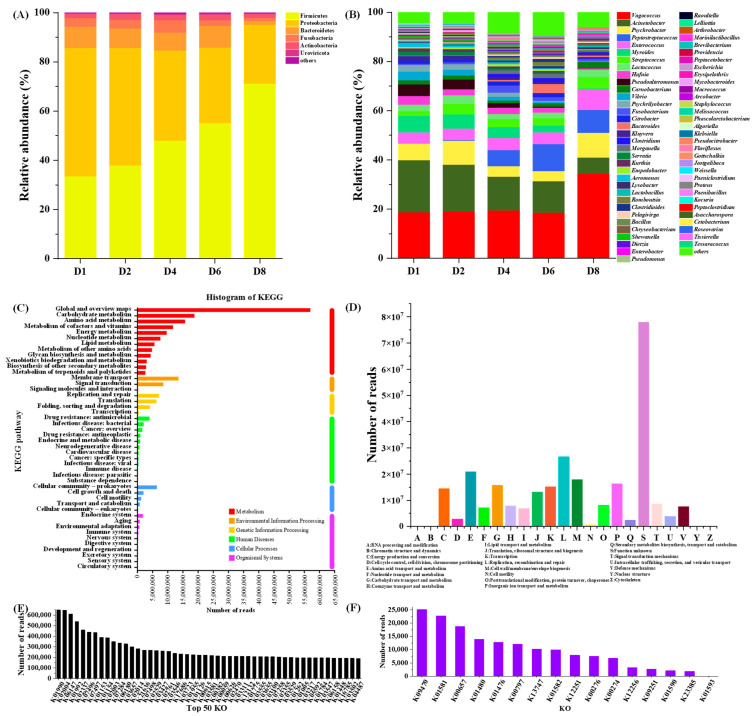
Metagenomic analysis of microbial community during fermentation of *Chouguiyu*. (**A**) Microbial taxonomic composition at the (**A**) phylum and (**B**) genus levels. Gene annotations of metagenome with the (**C**) KEGG and (**D**) COG databases. The (**E**) top 50 KO functions of the gene sequences and the (**F**) KO functions related to BA metabolism.

BAs are potential risks in *Chouguiyu* due to high concentrations of free amino acids and the complex microbial community. To investigate this, we analyzed the metagenome of the microbial community during *Chouguiyu* fermentation (Figure 1). According to the taxonomic analysis of obtained genes of the metagenome, Firmicutes and Proteobacteria were the main phyla and their total abundance accounted for over 85% during fermentation (Figure 1A). With increasing fermentation time, Firmicutes obviously increased while Proteobacteria obviously decreased, in accordance with the results in other fermented aquatic products [7,18], reflecting a successional pattern common to fermented fish. As shown in Figure 1B, there were a total of 66 microbial genera (relative abundance > 0.1%). *Vagococcus*, *Acinetobacter*, *Psychrobacter*, *Peptostreptococcus*, *Enterococcus*, *Myroides*, and *Streptococcus* were the dominant genera during the fermentation of *Chouguiyu*. Among these genera, *Acinetobacter* was the most abundant genus in the initial fermentation but was significantly inhibited thereafter. A similar trend was also found in *Myroides*. With an increase in fermentation time, the abundance of *Vagococcus*, *Peptostreptococcus*, and *Enterococcus* obviously increased, while *Psychrobacter* first increased and then decreased. *Vagococcus*, *Peptostreptococcus*, *Psychrobacter*, and *Enterococcus* became dominant at the end of fermentation.

**Figure 2:**
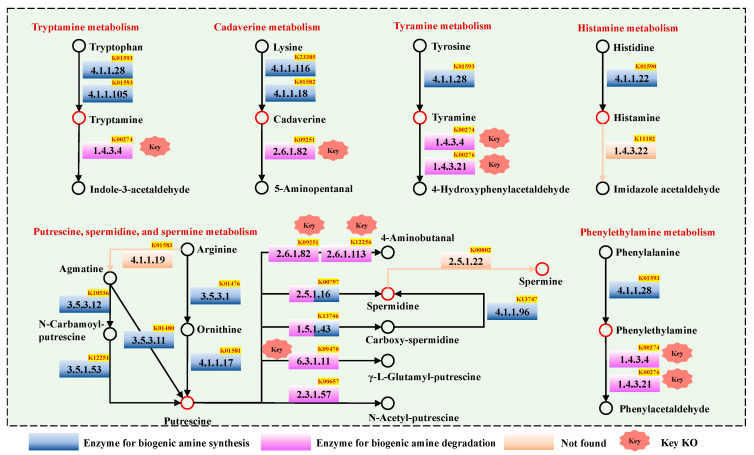
Biogenic amine metabolic pathways of microbial community in *Chouguiyu*.

In order to obtain gene annotations of the metagenome of the microbial community, the gene sequences were further annotated using the KEGG and COG databases. As shown in Figure 1C, the KEGG pathways mainly focused on metabolism, among which carbohydrate metabolism and amino acid metabolism had the highest abundance. As shown in Figure 1D, except for function unknown (S), most gene sequences are annotated as replication, recombination, and repair (L) and amino acid transport and metabolism (E). The high abundance of genes related to amino acid metabolism provided the basis for analyzing the metabolism process of BAs. KEGG orthology (KO) is a straight-homologous classification system that can group genes with similar sequences and functions and use the functions of known genes as the KO functions for cross-species annotation. All the gene sequences of the microbial community in *Chouguiyu* were totally annotated with 13,968 KO functions. The top 50 KO functions are shown in Figure 1E. K01990, K02004, K06147, and K01992 had the highest gene abundance during the fermentation of *Chouguiyu*. The KO functions related to BA metabolism were further identified (Figure 1F). A total of 16 KO functions were found in the metagenome of the microbial community. Among these, K00797, K01476, K01480, K01581, K01582, K01590, K01593, K10536, K12251, K13746, K13747, and K23385 were related to BA synthesis, while K00274, K00276, K00657, K00797, K09251, K09470, K12256, K13746, and K13747 contributed to BA degradation. K00797, K13746, and K13747 function in both synthesis and degradation pathways (Figure 2).

### 3.2. Enzymes Related to Biogenic Amine Metabolism in Chouguiyu Based on KO Functions

In order to fully understand the microbial metabolism mechanism and related enzymes of BAs in *Chouguiyu*, the BA metabolic pathways in *Chouguiyu* were constructed according to KO functions (Figure 2, Tables S2 and S3). For tryptamine metabolism, tryptamine was produced from tryptophan by L-tryptophan decarboxylase (EC 4.1.1.28 and EC 4.1.1.105) in K01593, while monoamine oxidase (EC 1.4.3.4) in K00274 could degrade tryptamine. Cadaverine was mainly produced from lysine by D-lysine decarboxylase (EC 4.1.1.116) in K23385 and lysine decarboxylase (EC 4.1.1.18) in K01582, while putrescine aminotransferase (EC 2.6.1.82) in K09251 could degrade cadaverine into 5-aminopentanal. Tyramine was obtained from tyrosine using L-tryptophan decarboxylase (EC 4.1.1.28) in K01593 and could be degraded by monoamine oxidase (EC 1.4.3.4) in K00274 and primary-amine oxidase (EC 1.4.3.21) in K00276. Histamine was generated from histidine by histidine decarboxylase (EC 4.1.1.22) in K01590, while no histamine-degrading enzymes were identified in *Chouguiyu*.

As one of the most abundant BAs, putrescine could be synthesized in a variety of ways. Arginine was first catalyzed by arginase (EC 3.5.3.1, K01476) into ornithine, which was further transferred into putrescine by ornithine decarboxylase (EC 4.1.1.17, K01581). This pathway was also the main putrescine-producing pathway in fermented foods [19]. Putrescine could also be synthesized through agmatine deiminase (EC 3.5.3.12) in K10536 and N-carbamoylputrescine amidase (EC 3.5.1.53) in K12251 or agmatinase (EC 3.5.3.11) in K01480 using agmatine as the substrate. Meanwhile, many enzymes contributed to the degradation of putrescine in *Chouguiyu*, including putrescine aminotransferase (EC 2.6.1.82) in K09251 and pyruvate transaminase (EC 2.6.1.113) in K12256 for 4-aminobutanal production, gamma-glutamylputrescine synthase (EC 6.3.1.11) in K09470 for γ-L-glutamyl-putrescine production, and diamine N-acetyltransferase (EC 2.3.1.57) in K00657 for N-acetyl-putrescine production.

In addition, putrescine was also catalyzed into spermidine by spermidine synthase (EC 2.5.1.16) in K00797 or was first degraded into carboxy-spermidine by carboxynorspermidine synthase (EC 1.5.1.43) in K13746 and transferred into spermidine by carboxynorspermidine decarboxylase (EC 4.1.1.96) in K13747. No spermidine-degrading enzyme, i.e., spermine-synthesizing enzyme, was observed in *Chouguiyu*.

Phenylethylamine was produced from phenylalanine by L-tryptophan decarboxylase (EC 4.1.1.28) in K01593. The enzymes for phenylethylamine degradation were the same as those for tyramine degradation, resulting in the production of phenylacetaldehyde.

### 3.3. Microbial Genus Related to Biogenic Amine Metabolism in Chouguiyu Based on KO

#### 3.3.1. Microbial Genera for BA Synthesis

**Figure 3:**
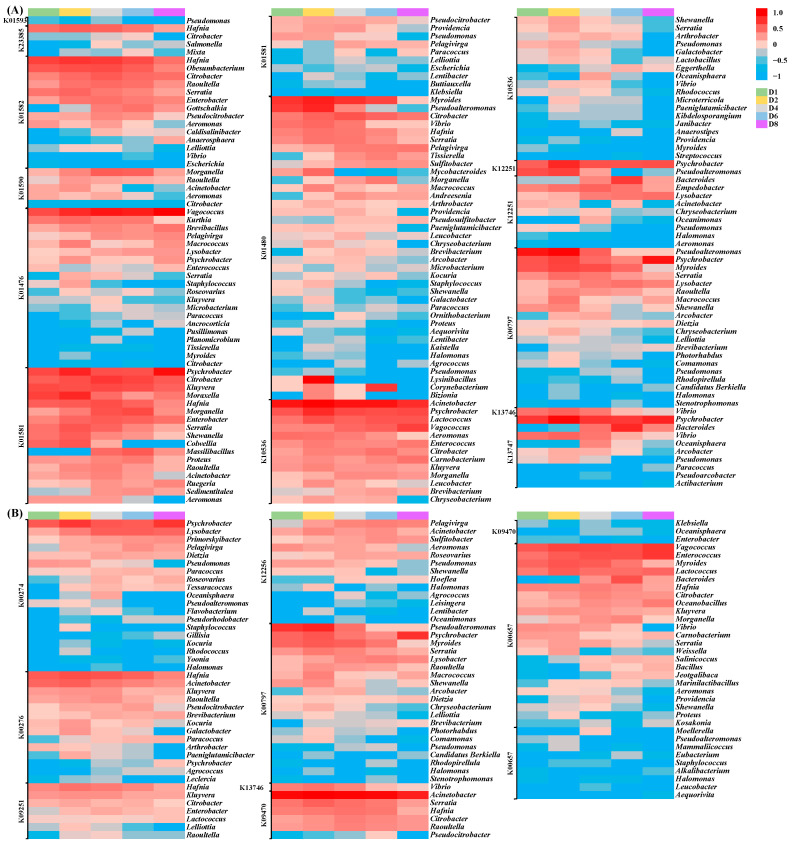
Abundance of enzymes for the (**A**) synthesis and (**B**) degradation of biogenic amines from the microbial community in *Chouguiyu*.

After analyzing the KO abundance, there were a total of 111 microbial genera related to BA metabolism (Figure 3). Among these, 91 microbial genera possessed BA synthesis ability (Figure 3A). *Pseudomonas* was the only microbial genus that possessed L-tryptophan decarboxylase (K01593), which was the most important enzyme for producing tryptamine, tyramine, and phenylethylamine. The gene abundance of *Pseudomonas* first increased and then decreased to none. Similarly, previous studies found that *Pseudomonas* was one of the main microorganisms to produce tryptamine [20], tyramine [5], and phenylethylamine [21].

The lysine decarboxylase in *Hafnia* can effectively produce cadaverine [22]. In this study, *Hafnia* was the most important microbial genus for the production of D-lysine decarboxylase (K23385) and lysine decarboxylase (K01582) for cadaverine synthesis. In addition, a high abundance of lysine decarboxylase (K01582) was found in *Obesumbacterium*, *Citrobacter*, *Raoultella*, *Serratia*, and *Enterobacter*, contributing to abundant cadaverine synthesis. Similar results were found showing that the expression of the decarboxylase gene in *Serratia* [23], as well as *Citrobacter* and *Enterobacter* [24,25,26], was associated with cadaverine production in foods. There were five microbial genera related to histamine production, among which *Morganella* and *Raoultella* contained the most abundant histidine decarboxylase (K01590). The histamine-producing ability of *Morganella* [27] and *Raoultella* [28] has also been reported in other studies.

Putrescine was mainly produced by a two-step reaction of agmatinase (K01476) and ornithine decarboxylase (K01581). A total of 20 microbial genera were responsible for the production of agmatinase, and most arginase-producing genera increased along with the fermentation process. Among these, *Vagococcus*, *Kurthia*, *Brevibacillus*, and *Pelagivirga* were the main microbial genera for agmatinase production. More microbial genera (27 kinds) could produce ornithine decarboxylase, and *Psychrobacter*, *Citrobacter*, *Kluyvera*, and *Moraxella* were the main microbial genera. However, there were only six microbial genera that could produce agmatinase and ornithine decarboxylase together, including *Pelagivirga*, *Psychrobacter*, *Serratia*, *Kluyvera*, *Paracoccus*, and *Citrobacter*. For putrescine synthesis from agmatine, there were 38 microbial genera secreting agmatinase (K01480), among which *Myroides*, *Pseudoalteromonas*, *Citrobacter*, and *Vibrio* played an important role in enzyme secretion. Most agmatinase-producing genera with high abundance decreased in the late fermentation.

In addition, putrescine could also be produced by a two-step reaction of agmatine deiminase (K10536) and N-carbamoylputrescine amidase (K12251) from agmatine. A total of 31 and 11 microbial genera were responsible for the production of agmatine deiminase and N-carbamoylputrescine amidase. Most genera with a high abundance of agmatine deiminase production increased with the increasing fermentation time. *Acinetobacter*, *Psychrobacter*, *Lactococcus*, and *Vagococcus* were the main microbial genera for agmatine deiminase production, while *Psychrobacter*, *Pseudoalteromonas*, *Bacteroides*, and *Empedobacter* were the main microbial genera for N-carbamoylputrescine amidase. Only *Acinetobacter*, *Psychrobacter*, *Aeromonas*, *Chryseobacterium*, and *Pseudomonas* produced these two enzymes together. The pathways of putrescine production are more complicated than those of other BAs and many studies have reported that putrescine can be generated from similar microorganisms, such as *Enterobacter* [29], *Citrobacter* [10], *Proteus* [24,30], *Serratia* [31], *Aeromonas* [32], and *Lactococcus* [32].

A total of 20 microbial genera possessed a high abundance of spermidine synthase (K00797) to produce spermidine, among which *Pseudoalteromonas*, *Psychrobacter*, *Myroides*, *Serratia*, *Lysobacter*, *Raoultella*, *Macrococcus*, and *Shewanella* were the main microbial genera. Spermidine was also produced by carboxynorspermidine synthase (K13746) and carboxynorspermidine decarboxylase (K13747) from putrescine. However, only *Vibrio* could produce carboxynorspermidine synthase, while there were nine microbial genera that possessed the ability to produce carboxynorspermidine decarboxylase, such as *Psychrobacter*, *Bacteroides*, and *Vibrio*. Similarly, *Psychrobacter* [33], *Bacteroides* [34], *Serratia* [35], and *Shewanella* [36] showed greater capacity to produce spermidine. Spermidine was further degraded into spermine using spermine synthase. However, there was no microbial metabolic gene for spermine in *Chouguiyu*.

#### 3.3.2. Microbial Genera for BA Degradation

Besides the abundant microorganisms for the production of BAs, many microorganisms (75 microbial genera) possessed various enzymes related to the degradation of BAs in *Chouguiyu* (Figure 3B). In this study, a total of 73 microbial genera possessed BA degradation ability and 19 microbial genera were potentially able to produce monoamine oxidase (K00274) for tryptamine, tyramine, and phenylethylamine degradation, among which *Psychrobacter*, *Lysobacter*, *Primorskyibacter*, *Pelagivirga*, and *Dietzia* possessed the highest abundance and increased along with the fermentation process. Tyramine and phenylethylamine could also be degraded by primary-amine oxidase (K00276). A total of 14 microbial genera generated primary-amine oxidase. *Hafnia*, *Acinetobacter*, *Kluyvera*, and *Raoultella* were the main microbial genera to produce the enzyme. Most genera with a high abundance of primary-amine oxidase production decreased with the prolongation of fermentation time. A total of 7 microbial genera played an important role in secreting putrescine aminotransferase (K09251) for cadaverine and putrescine degradation, and *Hafnia*, *Kluyvera*, *Citrobacter*, *Enterobacter*, and *Lactococcus* possessed the highest abundance. *Lactococcus* has previously been found to possess the enzyme activities responsible for BA degradation in cheese [37]. In addition, putrescine could be degraded by pyruvate transaminase (K12256), spermidine synthase (K00797), carboxynorspermidine synthase (K13746), gamma-glutamylputrescine synthase (K09470), and diamine N-acetyltransferase (K00657). Pyruvate transaminase was produced from 13 microbial genera, such as *Pelagivirga*, *Acinetobacter*, *Sulfitobacter*, and *Aeromonas*. The microbial genera for putrescine degradation using spermidine synthase and carboxynorspermidine synthase were the same as those for spermidine synthesis. There were nine genera that produced gamma-glutamylputrescine synthase, among which *Acinetobacter*, *Serratia*, *Hafnia*, *Citrobacter*, and *Raoultella* were the main genera. More genera (32 kinds) had the ability to produce diamine N-acetyltransferase, and *Vagococcus*, *Enterococcus*, *Myroides*, and *Lactococcus* were the main genera with high abundance. It was reported that *Enterococcus*’s multicopper oxidases could degrade phenylethylamine, putrescine, histamine, and tyramine [25]. Interestingly, some microbial genera possessed various enzymes for the degradation of various BAs in *Chouguiyu*, such as *Acinetobacter*, *Aeromonas*, *Arthrobacter*, *Hafnia*, *Lysobacter*, *Raoultella*, and *Pseudomonas*. Similar results were found showing that *Arthrobacter* was widely used to produce various amine oxidases for phenylethylamine, tyramine, and histamine degradation [38].

Interestingly, huge differences in microbial composition were found using total genes of the metagenome (Figure 1B) and genes related to BA metabolism (Figure 3). It was more scientific to analyze the mechanism of BA production and degradation based on functional gene abundance.

### 3.4. Change in Biogenic Amines During Fermentation of Chouguiyu

**Figure 4:**
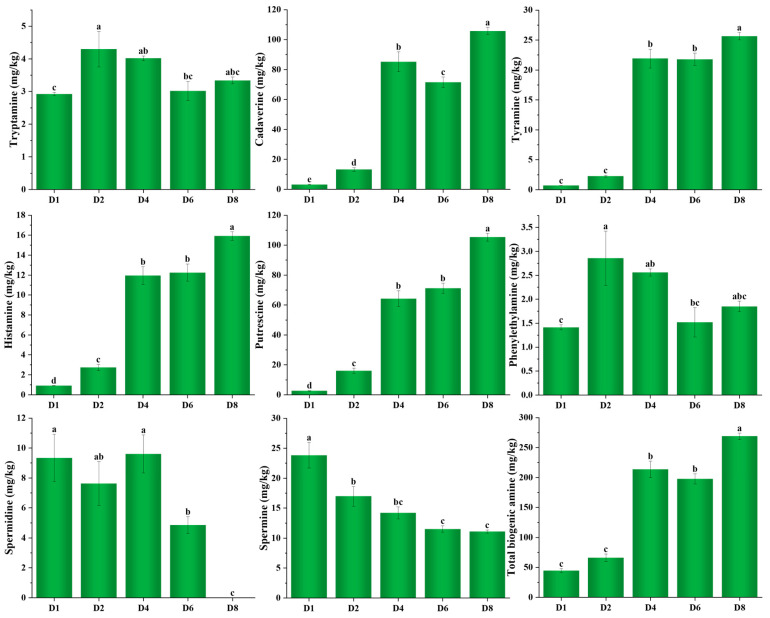
Change in biogenic amines during fermentation of *Chouguiyu*. Bars with different lowercase letters differ at *p* < 0.05 by one-way analysis of variance and Tukey test.

In order to clarify the actual influence of microbial genera on BAs of *Chouguiyu*, the change rules of eight BAs in *Chouguiyu* at different fermentation time are shown in Figure 4. As the fermentation time increased, tryptamine and phenylethylamine first increased and then decreased, and all reached maximums at 2 d of 4.30 and 2.86 mg/kg, respectively. Interestingly, the gene abundance of K01593 in *Pseudomonas* was low in *Chouguiyu* (Figure 3), resulting in the relatively low content of these two BAs. Cadaverine, tyramine, histamine, and putrescine markedly increased with the increasing fermentation time, while spermidine and spermine decreased. Histamine is considered the most toxic BA in fermented foods [7]. Worldwide, the legal limit for histamine in aquatic products varies by country and product type [39]. Among these, the minimum limit is 30 mg/kg and the United States suggests that the histamine safe limit in aquatic products is 50 mg/kg [40]. In this study, the histamine content reached a maximum of 15.91 mg/kg after fermentation for 8 d, indicating the safety of *Chouguiyu*. And the abundance of histidine decarboxylase genes decreased in the late fermentation, which might be an important reason for the low production of histamine. Tyramine also shows high toxicity, and its excessive intake can cause headaches and high blood pressure [24]. In this study, the maximal tyramine content was 25.65 mg/kg after 8 d fermentation.

Cadaverine and putrescine, despite being non-toxic in themselves, can inhibit the metabolic enzyme activity of histamine and tyramine (such as monoamine oxidase and diamine oxidase), therefore enhancing the toxicity of histamine and tyramine [31]. In this study, cadaverine and putrescine were the highest BAs in *Chouguiyu* at D8, reaching their maximums of 105.73 mg/kg and 105.30 mg/kg, respectively. The smelly flavor of these two BAs might contribute to the unique flavor of *Chouguiyu*. Interestingly, the abundance of the cadaverine-producing genes in almost all microbial genera first increased and then decreased during fermentation of *Chouguiyu*. There were low contents of spermidine, spermine, tryptamine, and phenylethylamine in the late fermentation of *Chouguiyu*, especially spermidine, which was not even detected.

### 3.5. Relationship of Biogenic Amine and Metabolic Pathway in Chouguiyu

**Figure 5:**
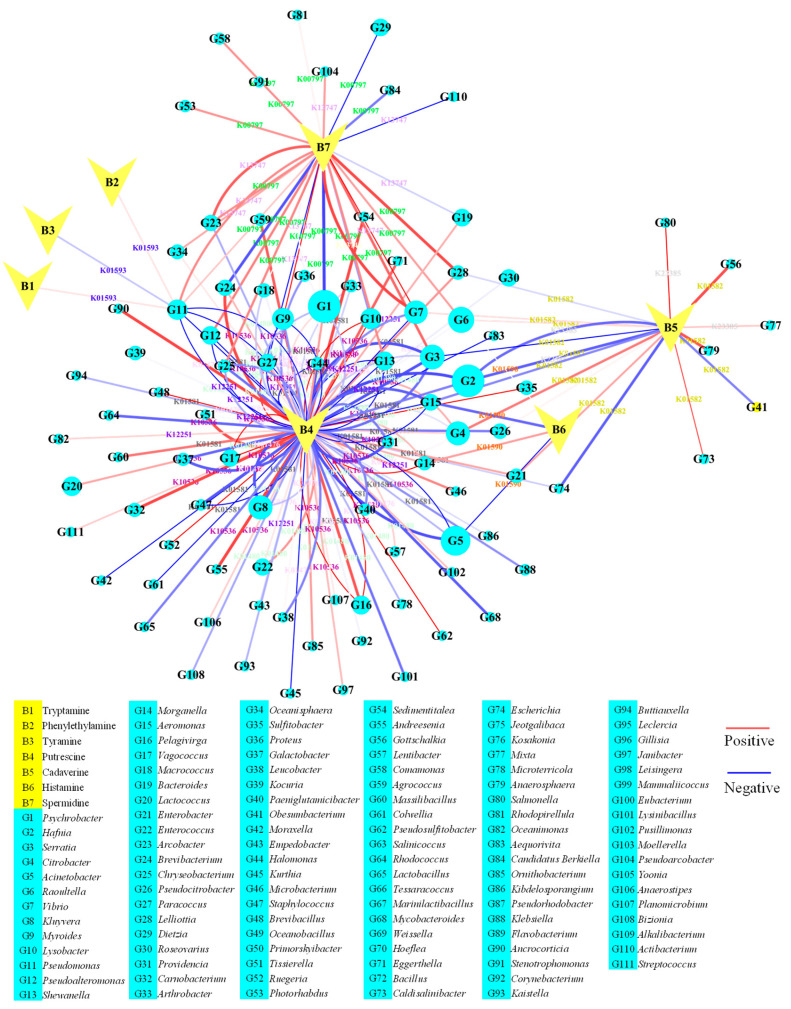
Correlation network map between microbial synthesis enzymes and biogenic amines. The red and blue lines, respectively, represent positive and negative correlation.

The relationship between microorganisms and their metabolites can be clearly visualized by correlation network maps [41,42]. To elucidate the microbial sources of biogenic amines (BAs), Pearson’s correlation between microbial synthesis enzymes and BAs was analyzed within each KO (Figure 5 and Figure S1 and Table S4). L-tryptophan decarboxylase (K01593) from *Pseudomonas* was positively correlated with tryptamine and phenylethylamine, while a negative correlation was observed with tyramine. *Pseudomonas* was similarly identified as an important genus for tryptamine production in correlative analyses [43]. Cadaverine levels were significantly positively correlated with D-lysine decarboxylase (K23385) from *Salmonella* but negatively correlated with the enzyme from *Hafnia*. Cadaverine was significantly positively correlated with lysine decarboxylase (K01582) from *Caldisalinibacter*, *Gottschalkia*, *Anaerosphaera*, and *Citrobacter* but was negatively correlated with that from *Serratia*, *Hafnia*, and *Escherichia*.

*Salmonella*, *Caldisalinibacter*, *Gottschalkia*, *Anaerosphaera*, and *Citrobacter* might play a major role in cadaverine synthesis. Similarly, cadaverine in *Dongbei Suancai* (fermented cabbage from Dongbei) was highly related to *Citrobacter* [9]. The change in putrescine was significantly positively correlated with arginase (K01476) from *Pelagivirga*, *Lysobacter*, *Vagococcus*, *Paracoccus*, and *Brevibacillus*, ornithine decarboxylase (K01581) from *Ruegeria*, *Sedimentitalea*, *Paracoccus*, and *Pelagivirga*, N-carbamoylputrescine amidase (K12251) from *Lysobacter*, and agmatinase (K01480) from *Pseudosulfitobacter*, *Pelagivirga*, *Sulfitobacter*, *Andreesenia*, *Tissierella*, and *Agrococcus*. The enhanced abundance of enzymes from these genera might play an important role in putrescine production.

Histamine was positively correlated with histidine decarboxylase (K01590) from *Morganella* and *Raoultella*, with *Morganella*—an established histamine-producing bacterium in seafood—being particularly notable [44]. Spermidine levels were significantly positively correlated with carboxynorspermidine decarboxylase (K13747) from *Vibrio* and *Arcobacter* and with spermidine synthase (K00797) from *Myroides*, *Lelliottia*, and *Serratia*. *Vibrio*, *Arcobacter*, *Myroides*, *Lelliottia*, and *Serratia* might be the major spermidine-synthesizing bacteria. Based on correlation analysis, *Serratia* was positively correlated with most of the BAs in soybean paste [26].

**Figure 6:**
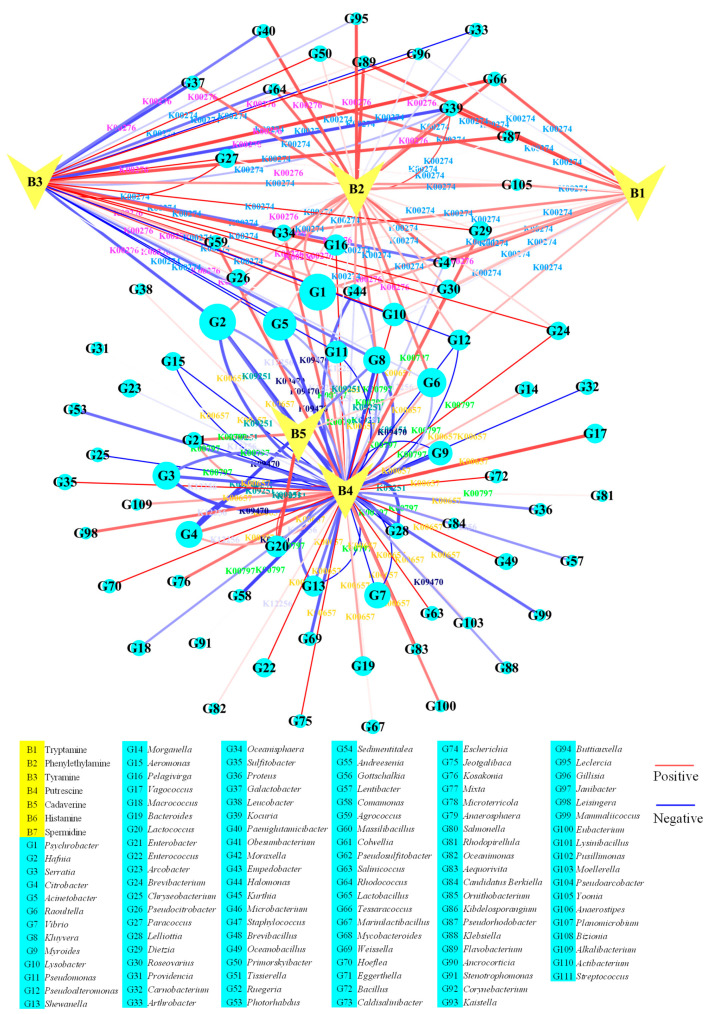
Correlation network map between microbial degradation enzymes and biogenic amines. The red and blue lines, respectively, represent positive and negative correlation.

For the correlation between BAs and their degradation enzymes from microorganisms in *Chouguiyu*, Pearson’s correlation between microbial degradation enzymes and BAs in each KO was analyzed (Figure 6 and Figure S2 and Table S5). Tryptamine levels were significantly positively correlated with monoamine oxidase (K00274) from *Kocuria*, *Flavobacterium*, and *Tessaracoccus*. Cadaverine was significantly positively correlated with putrescine aminotransferase (K09251) from *Lactococcus* but was negatively correlated with that from *Citrobacter* and *Lelliottia*. Declining *Citrobacter* and *Lelliottia* abundances correlated with cadaverine accumulation, suggesting reduced degradation capacity, and the decrease in *Lactococcus* was beneficial to the control of cadaverine accumulation. Tyramine levels were significantly positively correlated with monoamine oxidase from *Primorskyibacter*, *Lysobacter*, *Roseovarius*, *Dietzia*, *Paracoccus*, *Gillisia*, *Tessaracoccus*, *Pseudorhodobacter*, and *Pelagivirga* and positively related with primary-amine oxidase from *Brevibacterium*, *Agrococcus*, *Paracoccus*, and *Psychrobacter*. The increasing enzymes in these genera might contribute to the inhibition of tyramine accumulation. Conversely, tyramine concentration was significantly negatively correlated with monoamine oxidase from *Pseudomonas* and *Pseudoalteromonas* and with primary-amine oxidase from *Acinetobacter*, *Arthrobacter*, *Galactobacter*, *Kocuria*, and *Hafnia*, indicating their role in tyramine increase.

The change in putrescine was also significantly positively correlated with putrescine aminotransferase (K09251) from *Lactococcus*, pyruvate transaminase (K12256) from *Pelagivirga*, *Sulfitobacter*, *Roseovarius*, and *Hoeflea*, spermidine synthase (K00797) from *Lysobacter*, *Brevibacterium*, and *Dietzia*, and diamine N-acetyltransferase (K00657) from *Oceanobacillus*, *Bacillus*, *Enterococcus*, *Salinicoccus*, *Jeotgalibaca*, *Vagococcus*, and *Aequorivita*. These increasing enzymes in the above genera were important for the inhibition of putrescine in *Chouguiyu*. Meanwhile, putrescine was significantly negatively correlated with putrescine aminotransferase from *Citrobacter*, *Lelliottia*, *Kluyvera*, and *Hafnia*, pyruvate transaminase from *Pseudomonas*, *Aeromonas*, *Halomonas*, and *Shewanella*, spermidine synthase from *Pseudoalteromonas*, *Shewanella*, *Lelliottia*, *Myroides*, *Chryseobacterium*, and *Comamonas*, carboxynorspermidine synthase (K13746) from *Vibrio*, gamma-glutamylputrescine synthase (K09470) from *Enterobacter*, *Serratia*, *Acinetobacter*, and diamine N-acetyltransferase from *Vibrio*, *Aeromonas*, *Pseudoalteromonas*, *Carnobacterium*, *Kluyvera*, *Myroides*, *Hafnia*, *Serratia*, and *Weissella*. The decreasing enzymes in these genera contributed most to the increase in putrescine during fermentation of *Chouguiyu*.

The change in phenylethylamine was significantly positively correlated with monoamine oxidase from *Kocuria*, *Flavobacterium*, and *Tessaracoccus* and correlated with primary-amine oxidase from *Leclercia* and *Paeniglutamicibacter*. These microbial genera played a crucial role in the maintenance of phenylethylamine concentration. Meanwhile, the KOs of K09251, K00657, K00274, K12256, K09470, and K00276 were the major biological amine degradation pathways (Figure 2).

Interestingly, microbial degraders of tryptamine, tyramine, and phenylethylamine outnumbered producers, explaining their low concentrations. Conversely, putrescine and cadaverine accumulated due to scarce degraders (Figure 6).

### 3.6. Identification of Core Microorganisms for Biogenic Amine Degradation in Chouguiyu

**Figure 7:**
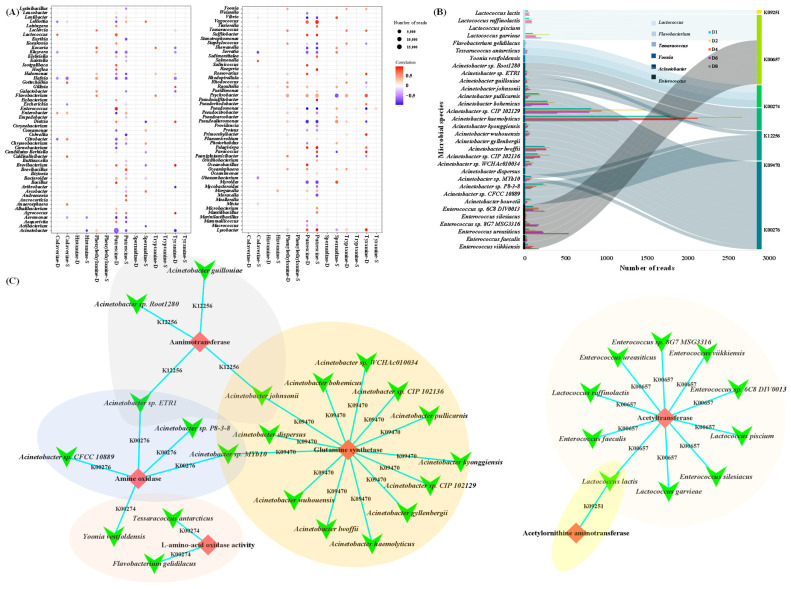
Core microbial genera for the degradation of biogenic amines. (**A**) Total abundance of functional enzymes related to biogenic amine synthesis and degradation in different microbial genera and their correlation with biogenic amines. (**B**) Core microbial species with the ability to degrade biogenic amines during the fermentation of *Chouguiyu*. (**C**) Relationship of core microbial species and enzymes related to biogenic amine degradation.

In order to more clearly focus on BA-degrading microbial species, the microorganisms associated with key degradation pathways are shown in Figure 7. In this study, the synthesis of cadaverine by microorganisms in *Chouguiyu* was higher than its degradation process, resulting from the increasing cadaverine concentration along with fermentation (Figure 7A). *Salmonella*, *Gottschalkia*, *Caldisalinibacter*, and *Anaerosphaera* contributed most to the production of cadaverine, while *Enterobacter* and *Lactococcus* were the main cadaverine-degrading microbial genera.

Among the histamine-producing genera, only *Morganella* showed a close relationship with histamine, and its low abundance resulted in the low content of histamine.

*Pseudomonas* was the only genus that was responsible for the synthesis of phenylethylamine, tryptamine, and tyramine, leading to the relatively low production of these BAs in *Chouguiyu*. More microbial genera were helpful in the degradation of phenylethylamine, among which *Psychrobacter*, *Hafnia*, *Lysobacter*, and *Acinetobacter* were the main degradation bacteria of phenylethylamine, while *Psychrobacter* and *Lysobacter* contributed most to tryptamine and tyramine degradation. *Vagococcus* and *Psychrobacter*, which possessed high enzyme abundance, were the main putrescine synthesis microorganisms, while *Vagococcus* and *Enterococcus* were beneficial for putrescine degradation. The high abundance of synthesis enzymes in *Psychrobacter*, *Pseudoalteromonas*, *Myroides*, and *Bacteroides* was important for spermidine synthesis, while there was no related enzyme for spermidine degradation. Interestingly, *Lactococcus*, *Flavobacterium*, *Tessaracoccus*, and *Yoonia* possessed only the BA degradation enzymes. Similarly to Korean jeotgal [45], *Chouguiyu*’s low-salt process permits diverse BA-producing microbes, but unlike jeotgal, it harbors unique degraders like *Yoonia*. In addition, *Acinetobacter* and *Enterococcus* had a much higher abundance of degradation enzymes than synthesis enzymes for BA metabolism.

The microbial species in the above genera for BA degradation were further explored (Figure 7B,C). For *Lactococcus*, a total of four microbial species had the BA degradation enzyme genes, among which *Lactococcus garvieae* possessed the most abundant putrescine degradation enzymes. *Lactococcus lactis* possessed both putrescine aminotransferase (K09251) and diamine N-acetyltransferase (K00657) for cadaverine and putrescine degradation, while *Lactococcus raffinolactis*, *Lactococcus piscium*, and *Lactococcus garvieae* only possessed diamine N-acetyltransferase for putrescine degradation. *Flavobacterium gelidilacus*, *Tessaracoccus antarcticus*, and *Yoonia vestfoldensis* were the only species that possessed monoamine oxidase (K00274) for tryptamine, tyrosine, and phenylalanine degradation in *Flavobacterium*, *Tessaracoccus*, and *Yoonia*, respectively. For *Acinetobacter*, a total of 19 microbial species possessed plenty of BA degradation genes, and they were observed to have a variety of putrescine, tyrosine, and phenylalanine degradation enzymes, including pyruvate transaminase (K12256), gamma-glutamylputrescine synthase (K09470), and primary-amine oxidase (K00276). Among these species, *Acinetobacter haemolyticus* possessed the highest enzyme activity for tyrosine and phenylalanine degradation. For *Enterococcus*, there were six microbial species that had putrescine degradation enzymes, i.e., diamine N-acetyltransferase. *Enterococcus ureasiticus* possessed more abundant enzymes than the other microbial species in *Enterococcus*.

It can be concluded that *Lactococcus garvieae*, *Flavobacterium gelidilacus*, *Tessaracoccus antarcticus*, *Yoonia vestfoldensis*, *Acinetobacter haemolyticus*, and *Enterococcus ureasiticus* were the main microbial species for BA degradation during the fermentation of *Chouguiyu*, which could be isolated as the potential functional strains for the degradation of BAs in fermented foods. According to the above results, the degradation of undesirable BAs in *Chouguiyu* could be reduced and isolated strains could be applied in fermented foods like fish sauce or cheese to reduce BA risks.

## 4. Conclusions

Based on KO analysis, there were 91 BA-synthesizing microbial genera, among which *Psychrobacter*, *Vagococcus*, *Pseudoalteromonas*, *Hafnia*, *Citrobacter*, and *Myroides* were the main genera, especially for putrescine synthesis. Seventy-five genera possessed degradation enzymes, primarily *Acinetobacter*, *Psychrobacter*, *Vagococcus*, *Pseudoalteromonas*, *Enterococcus*, *Myroides*, and *Hafnia*, especially for putrescine. After determination of BAs, high concentrations of cadaverine and putrescine were observed in *Chouguiyu*, while histamine and tyramine exhibited relatively low concentrations, and these four BAs increased with the increasing fermentation time, similar to KO analysis. The correlation networks revealed *Lactococcus*, *Flavobacterium*, *Tessaracoccus*, and *Yoonia* as non-producers but potential BA degraders. *Acinetobacter* and *Enterococcus* possessed more abundant degradation enzymes than synthesis enzymes. Amine oxidase from *Acinetobacter*, as well as diamine N-acetyltransferase from *Enterococcus* and *Lactococcus*, played the main role in biogenic amine degradation in *Chouguiyu*. *Lactococcus garvieae*, *Flavobacterium gelidilacus*, *Tessaracoccus antarcticus*, *Yoonia vestfoldensis*, *Acinetobacter haemolyticus*, and *Enterococcus ureasiticus* were the main microbial species for BA degradation and could be isolated as the potential functional strains for the degradation of BAs in fermented foods.

## Data Availability

The original contributions presented in this study are included in the article/Supplementary Material. Further inquiries can be directed to the corresponding authors.

## Supplementary Materials

The following supporting information can be downloaded at: https://www.mdpi.com/article/10.3390/foods14162863/s1, Table S1: Standard curves of different biogenic amine; Figure S1: Correlation heatmap between microbial synthesis enzymes and biogenic amines in each KO; Figure S2: Correlation heatmap between microbial degradation enzymes and biogenic amines in each KO. Table S2: Enzymes and relative abundance of reads involved in biogenic amine production in *Chouguiyu*; Table S3: Enzymes and relative abundance of reads involved in biogenic amine degradation in *Chouguiyu*; Table S4: Correlations and *p*-value between synthesis genus and biogenic amine in *Chouguiyu.* Table S5: Correlations and *p*-value between degradation genus and biogenic amine in *Chouguiyu.*

## References

[1] Dabade D.S., Jacxsens L., Miclotte L., Abatih E., Devlieghere F., De Meulenaer B. (2021). Survey of multiple biogenic amines and correlation to microbiological quality and free amino acids in foods. Food Control.

[2] Turna N.S., Chung R., Mcintyre L. (2024). A review of biogenic amines in fermented foods: Occurrence and health effects. Heliyon.

[3] Li Y., Li W., Li C., Li L., Yang D., Wang Y., Chen S., Wang D., Wu Y. (2023). Novel insight into flavor and quality formation in naturally fermented low-salt fish sauce based on microbial metabolism. Food Res. Int..

[4] Jiang W., Li C., Xu B., Dong X., Ma N., Yu J., Wang D., Xu Y. (2014). Halomonas shantousis sp. nov., a novel biogenic amines degrading bacterium isolated from Chinese fermented fish sauce. Antonie Leeuwenhoek.

[5] Moon J.S., Cho S.K., Choi H.Y., Kim J.E., Kim S., Cho K., Han N.S. (2010). Isolation and characterization of biogenic amine-producing bacteria in fermented soybean pastes. J. Microbiol..

[6] Li C., Cui Q., Li L., Huang H., Chen S., Zhao Y., Wang Y. (2024). Formation and improvement mechanism of physical property and volatile flavor of fermented tilapia surimi by newly isolated lactic acid bacteria based on two dimensional correlation networks. Food Chem..

[7] Zhao Y., Wang Y., Li C., Li L., Yang X., Wu Y., Chen S., Zhao Y. (2021). Novel insight into physicochemical and flavor formation in naturally fermented tilapia sausage based on microbial metabolic network. Food Res. Int..

[8] Sang X., Ma X., Hao H., Bi J., Zhang G., Hou H. (2020). Evaluation of biogenic amines and microbial composition in the Chinese traditional fermented food grasshopper sub shrimp paste. LWT-Food Sci. Technol..

[9] Ye H., Lang X., Ji Y., Li S., Xin N., Meng X., Zhang T., Shen X., Zhao C. (2021). The interaction between Lactobacillus plantarum SC-5 and its biogenic amine formation with different salt concentrations in Chinese Dongbei Suancai. Food Res. Int..

[10] Li R., Zheng M., Zheng M., Cai R., Cui X., Wang Y., Jiang X., Xu C. (2022). Metagenomic analysis reveals the linkages between bacteria and the functional enzymes responsible for potential ammonia and biogenic amine production in alfalfa silage. J. Appl. Microbiol..

[11] Yang D., Li C., Li L., Wang Y., Wu Y., Chen S., Zhao Y., Wei Y., Wang D. (2022). Novel insight into the formation mechanism of umami peptides based on microbial metabolism in Chouguiyu, a traditional Chinese fermented fish. Food Res. Int..

[12] Hu M., Dong J., Tan G., Li X., Zheng Z., Li M. (2021). Metagenomic insights into the bacteria responsible for producing biogenic amines in sufu. Food Microbiol..

[13] Yang Z., Liu S., Lv J., Sun Z., Xu W., Ji C., Liang H., Li S., Yu C., Lin X. (2020). Microbial succession and the changes of flavor and aroma in Chouguiyu, a traditional Chinese fermented fish. Food Biosci..

[14] Shen Y., Wu Y., Wang Y., Li L., Li C., Zhao Y., Yang S. (2021). Contribution of autochthonous microbiota succession to flavor formation during Chinese fermented mandarin fish (*Siniperca chuatsi*). Food Chem..

[15] Yang D., Li C., Li L., Chen S., Hu X., Xiang H. (2022). Taste mechanism of umami peptides from Chinese traditional fermented fish (*Chouguiyu*) based on molecular docking using umami receptor T1R1/T1R3. Food Chem..

[16] Yang D., Li L., Li C., Chen S., Deng J., Yang S. (2022). Formation and inhibition mechanism of novel angiotensin I converting enzyme inhibitory peptides from *Chouguiyu*. Front. Nutr..

[17] Li Y., Cui L., Du F., Han X., Li J. (2021). Impacts of ε-polylysine hydrochloride with thymol on biogenic amines formation and biochemical changes of squid (*Illexargentinus*). J. Food Process. Preserv..

[18] Wang Y., Li C., Zhao Y., Li L., Yang X., Wu Y., Chen S., Cen J., Yang S., Yang D. (2020). Novel insight into the formation mechanism of volatile flavor in Chinese fish sauce (Yu-lu) based on molecular sensory and metagenomics analyses. Food Chem..

[19] Romano A., Ladero V., Alvarez M.A., Lucas P.M. (2014). Putrescine production via the ornithine decarboxylation pathway improves the acid stress survival of *Lactobacillus brevis* and is part of a horizontally transferred acid resistance locus. Int. J. Food Microbiol..

[20] Kosma I., Badeka A. (2021). Determination of six underivatized biogenic amines by LC-MS/MS and study of biogenic amine production during trout (*Salmo trutta*) storage in ice. Food Addit. Contam. Part A.

[21] Geornaras I., Dykes G.A., von Holy A. (1995). Biogenic amine formation by poultry-associated spoilage and pathogenic bacteria. Lett. Appl. Microbiol..

[22] Wang C., Zhang K., Zhongjun C., Cai H., Honggui W., Ouyang P. (2015). Directed evolution and mutagenesis of lysine decarboxylase from *Hafnia alvei* AS1.1009 to improve its activity toward efficient cadaverine production. Biotechnol. Bioprocess Eng..

[23] Bubelova Z., Bunka F., Tatakova M., Stajnochova K., Purevdorj K., Bunkova L. (2015). Effects of temperature, pH and NaCl content on in vitro putrescine and cadaverine production through the growth of Serratia marcescens CCM 303. J. Environ. Sci. Health Part B.

[24] Greif G., Greifova M., Karovicova J. (2006). Effects of NaCl concentration and initial pH value on biogenic amine formation dynamics by *Enterobacter spp.* bacteria in model conditions. J. Food Nutr. Res..

[25] Li B., Wang Y., Xue L., Lu S. (2021). Heterologous expression and application of multicopper oxidases from *Enterococcus spp.* for degradation of biogenic amines. Protein Pept. Lett..

[26] Li S., Du X., Feng L., Mu G., Tuo Y. (2021). The microbial community, biogenic amines content of soybean paste, and the degradation of biogenic amines by *Lactobacillus plantarum* HM24. Food Sci. Nutr..

[27] Podeur G., Dalgaard P., Leroi F., Prévost H., Emborg J., Martinussen J., Hansen L.H., Pilet M. (2015). Development of a real-time PCR method coupled with a selective pre-enrichment step for quantification of Morganella morganii and Morganella psychrotolerans in fish products. Int. J. Food Microbiol..

[28] Lin C., Kung H., Lin C., Tsai H., Tsai Y. (2016). Histamine production by Raoultella ornithinolytica in mahi-mahi meat at various storage temperatures. J. Food Drug Anal..

[29] Lavizzari T., Breccia M., Bover-Cid S., Vidal-Carou M.C., Veciana-Nogues M.T. (2010). Histamine, cadaverine, and putrescine produced in vitro by Enterobacteriaceae and Pseudomonadaceae isolated from spinach. J. Food Prot..

[30] Helinck S., Perello M., Deetae P., de Revel G., Spinnler H. (2013). Debaryomyces hansenii, Proteus vulgaris, Psychrobacter sp. and Microbacterium foliorum are able to produce biogenic amines. Dairy Sci. Technol..

[31] De Filippis F., Pennacchia C., Di Pasqua R., Fiore A., Fogliano V., Villani F., Ercolini D. (2013). Decarboxylase gene expression and cadaverine and putrescine production by Serratia proteamaculans in vitro and in beef. Int. J. Food Microbiol..

[32] Zhang Y., Li D., Lv J., Li Q., Kong C., Luo Y. (2017). Effect of cinnamon essential oil on bacterial diversity and shelf-life in vacuum-packaged common carp (*Cyprinus carpio*) during refrigerated storage. Int. J. Food Microbiol..

[33] Kämpfer P., Jerzak L., Wilharm G., Golke J., Busse H., Glaeser S.P. (2015). *Psychrobacter ciconiae* sp. nov., isolated from white storks (*Ciconia ciconia*). Int. J. Syst. Evol. Microbiol..

[34] Sakanaka M., Sugiyama Y., Nara M., Kitakata A., Kurihara S. (2018). Functional analysis of arginine decarboxylase gene *speA* of *Bacteroides dorei* by markerless gene deletion. FEMS Microbiol. Lett..

[35] Chaves López C., De Angelis M., Martuscelli M., Serio A., Paparella A., Suzzi G. (2006). Characterization of the Enterobacteriaceae isolated from an artisanal Italian ewe’s cheese (*Pecorino Abruzzese*). J. Appl. Microbiol..

[36] Wang S., Liang H., Liu L., Jiang X., Wu S., Gao H. (2020). Promiscuous enzymes cause biosynthesis of diverse siderophores in Shewanella oneidensis. Appl. Environ. Microbiol..

[37] Guarcello R., De Angelis M., Settanni L., Formiglio S., Gaglio R., Minervini F., Moschetti G., Gobbetti M. (2016). Selection of amine-oxidizing dairy lactic acid bacteria and identification of the enzyme and gene involved in the decrease of biogenic amines. Appl. Environ. Microbiol..

[38] Lee J., Kim Y. (2013). Characterization of amine oxidases from *Arthrobacter aurescens* and application for determination of biogenic amines. World J. Microbiol. Biotechnol..

[39] DeBeeR J., Bell J.W., Nolte F., Arcieri J., Correa G. (2021). Histamine Limits by Country: A Survey and Review. J. Food Prot..

[40] FDA (2021). Fish and Fishery Products Hazards and Controls Guidance.

[41] Li C., Li W., Li L., Chen S., Wu Y., Qi B. (2022). Microbial community changes induced by a newly isolated salt-tolerant *Tetragenococcus muriaticus* improve the volatile flavor formation in low-salt fish sauce. Food Res. Int..

[42] Cui Q., Li L., Huang H., Yang Y., Chen S., Li C. (2024). Novel insight into the formation and improvement mechanism of physical property in fermented tilapia sausage by cooperative fermentation of newly isolated lactic acid bacteria based on microbial contribution. Food Res. Int..

[43] Yu Y., Li L., Xu Y., An K., Shi Q., Yu Y., Xu Z. (2021). Evaluation of the relationship among biogenic amines, nitrite and microbial diversity in fermented mustard. Molecules.

[44] Wang D., Li C., Pan C., Wang Y., Xiang H., Feng Y., Yang X., Chen S., Zhao Y., Wu Y. (2022). Antimicrobial activity and mechanism of action of oregano essential oil against *Morganella psychrotolerans* and potential application in tuna. LWT-Food Sci. Technol..

[45] Jung M.Y., Kim T., Lee C., Kim J.Y., Song H.S., Kim Y.B., Ahn S.W., Kim J.S., Roh S.W., Lee S.H. (2018). Role of jeotgal, a Korean traditional fermented fish sauce, in microbial dynamics and metabolite profiles during kimchi fermentation. Food Chem..

