# Supplementary material for: Novel Insight into Metabolism Mechanism of Biogenic Amines During Fermentation of Chinese Traditional Fermented Mandarin Fish (Chouguiyu) Based on Metabolism Pathway and Correlation Network
Source: Foods. 2025 Aug 18;14(16):2863. doi: 10.3390/foods14162863 (PMC12385225; doi:10.3390/foods14162863)
Supplement: Supplementary file 1 [file foods-14-02863-s001.zip › foods-3747639-supplementary.pdf]

Supplementary Data

Novel insight into metabolism mechanism of biogenic amines during fermentation of Chinese traditional fermented mandarin fish (*Chouguiyu*) based on metabolism pathway and correlation network

Jun Li[1], Daqiao Yang[2, 3, *], Yongqiang Zhao[2], Di Wang[2], Hui Huang[2], Chunsheng Li[2, *]

1 Guangdong Provincial Key Laboratory of Lingnan Specialty Food Science and Technology, Key Laboratory of Green Processing and Intelligent Manufacturing of Lingnan Specialty Food, Ministry of Agriculture and Rural, College of Light Industry and Food, Zhongkai University of Agriculture and Engineering, Guangzhou 510225, China; lijun@zhku.edu.cn (J.L.)

2 Key Laboratory of Aquatic Product Processing, Ministry of Agriculture and Rural Affairs, National R&D Center for Aquatic Product Processing, South China Sea Fisheries Research Institute, Chinese Academy of Fishery Sciences, Guangzhou 510300, China; zhaoyq@scsfri.ac.cn (Y.Z.); wangdi@scsfri.ac.cn (D.W.); huanghuigd@aliyun.com (H.H.); lichunsheng@scsfri.ac.cn (C.L.)

3 College of Life Sciences, Linyi University, Linyi 276000, China; yangdaqiao233@163.com (D.Y.);

* Correspondence: lichunsheng@scsfri.ac.cn; yangdaqiao233@163.com; Tel.: +86 20-89020911 (C.L.)

Table S1. Standard curves of different biogenic amine.

Biogenic Amines	Standard Curve	R^2
Tryptamine	y=11173x-3351.8	0.999
Phenylethylamine	y=10679.3x+1033.4	0.999
Putrescine	y=21231.9x+2663.11	0.999
Cadaverine	y=20436.5x-67.382	0.999
Histamine	y=15777x-1132.5	0.999
Tyramine	y=18982.9x-1712.6	0.999
Spermidine	y=17686.7x+29644.7	0.999
Spermine	y=16377.5x-1010.7	0.999

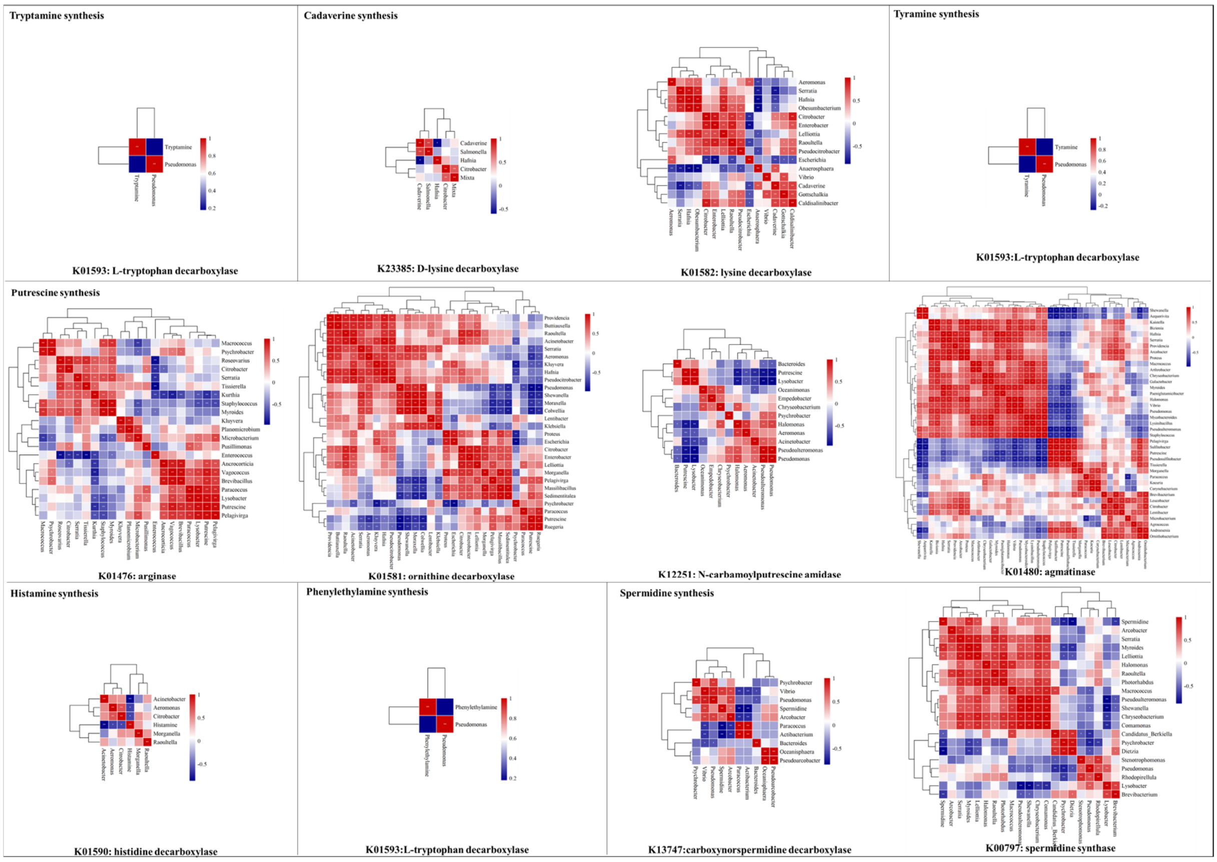

Figure S1. Correlation heatmap between microbial synthesis enzymes and biogenic amines in each KO.

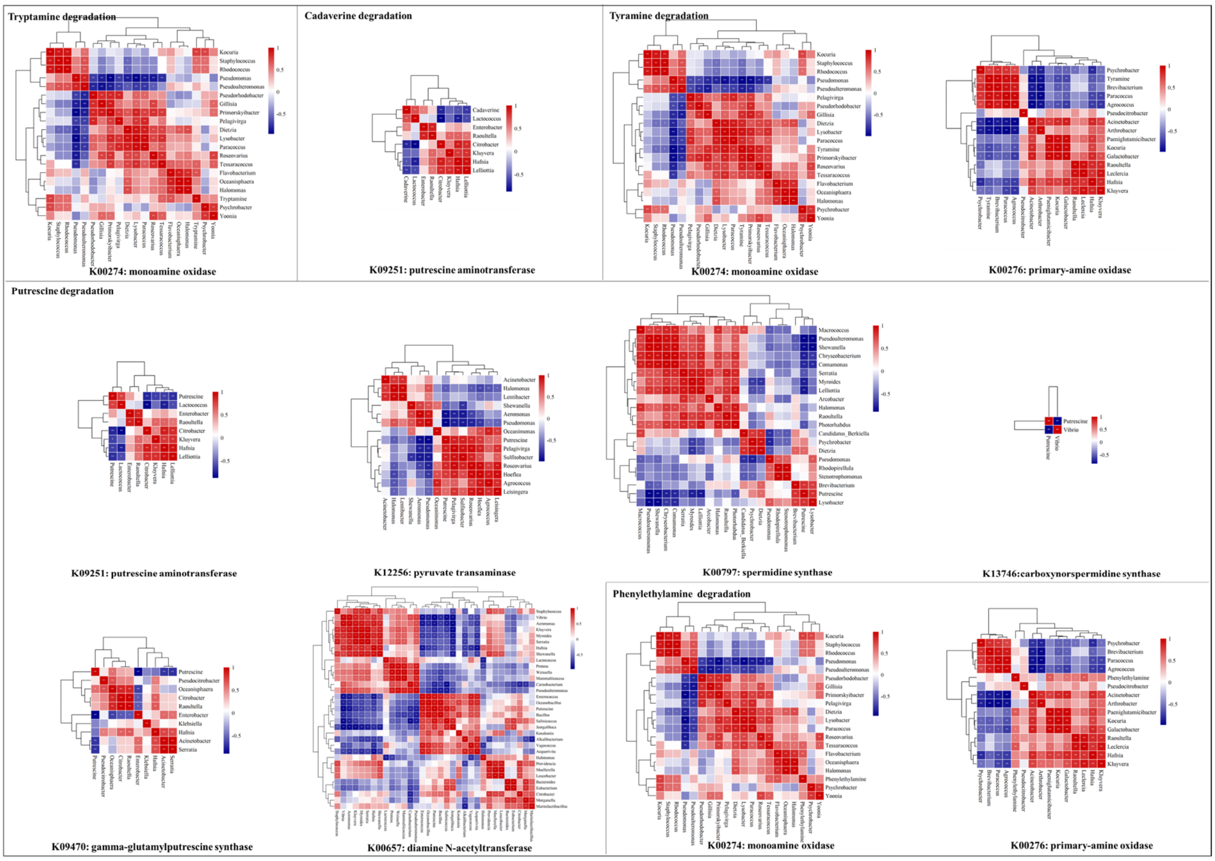

Figure S2. Correlation heatmap between microbial degradation enzymes and biogenic amines in each KO.

Table S2. Enzymes and relative abundance of reads involved in biogenic amine production in *Chouguiyu.*

KO	Substrate	Enzyme Description	Enzyme Code	D1	D2	D4	D6	D8
K00797	Putrescine	spermidine synthase	2.5.1.16	2210	3452	1306	716	1636
K01476	Arginine	arginase	3.5.3.1	726	1498	1742	1536	2876
K01480	Agmatine	agmatinase	3.5.3.11	2070	3496	2344	1644	784
K01581	Ornithine	ornithine decarboxylase	4.1.1.17	2904	4552	3850	3066	2890
K01582	Lysine	lysine decarboxylase	4.1.1.18	1284	1822	2058	1538	834
K01590	Histidine	histidine decarboxylase	4.1.1.22	142	270	384	296	94
K01593	Tryptophan/Tyrosine/Phenylalanine	L-tryptophan decarboxylase	4.1.1.28\4.1.1.105	4	2	6	0	0
K10536	Agmatine	agmatine deiminase	3.5.3.12	2500	4320	3092	3082	2574
K13746	Putrescine	carboxynorspermidine synthase	1.5.1.43	162	232	138	30	14
K13747	Carboxy-spermidine	carboxynorspermidine decarboxylase	4.1.1.96	808	1902	1108	1498	1072
K23385	Lysine	D-lysine decarboxylase	4.1.1.116	184	266	260	184	66
K12251	N-carbamoylputrescine	N-carbamoylputrescine amidase	3.5.1.53	822	1628	922	1214	668

Table S3. Enzymes and relative abundance of reads involved in biogenic amine degradation in *Chouguiyu.*

KO	Substrate	Enzyme Description	Enzyme Code	D1	D2	D4	D6	D8
K00274	Tryptamine/Tyramine	monoamine oxidase	1.4.3.4	660	1576	1248	1150	1804
K00276	Tyramine	primary-amine oxidase	1.4.3.21	996	1500	1108	790	440
K00657	Putrescine	diamine N-acetyltransferase	2.3.1.57	1790	2924	2952	2896	3238
K00797	Putrescine	spermidine synthase	2.5.1.16	2212	3462	1310	716	1640
K09251	Cadaverine	putrescine aminotransferase	2.6.1.82	276	452	386	278	172
K09470	Putrescine	gamma-glutamylputrescine synthase	6.3.1.11	4110	6364	4878	4256	2246
K12256	Putrescine	pyruvate transaminase	2.6.1.113	284	426	486	490	342
K13746	Putrescine	carboxynorspermidine synthase	1.5.1.43	162	232	138	30	14

Table S4. Correlations and p-value between synthesis genus and biogenic amine in *Chouguiyu*.

Genus	BAs	Correlation	*p*-Value	KO Source
Pseudomonas	Tryptamine	0.18	5.30E-01	K01593
Pseudomonas	Phenylethylamine	0.18	5.30E-01	K01593
Pseudomonas	Tyramine	-0.28	3.16E-01	K01593
Vagococcus	Putrescine	0.91	1.98E-06	K10536
Morganella	Putrescine	0.78	6.89E-04	K10536
Brevibacterium	Putrescine	0.73	1.88E-03	K10536
Carnobacterium	Putrescine	0.73	1.97E-03	K10536
Eggerthella	Putrescine	0.64	1.09E-02	K10536
Enterococcus	Putrescine	0.45	8.85E-02	K10536
Lactococcus	Putrescine	0.39	1.47E-01	K10536
Citrobacter	Putrescine	0.35	1.98E-01	K10536
Janibacter	Putrescine	0.33	2.33E-01	K10536
Anaerostipes	Putrescine	0.26	3.59E-01	K10536
Streptococcus	Putrescine	0.26	3.59E-01	K10536
Oceanisphaera	Putrescine	0.16	5.67E-01	K10536
Kibdelosporangium	Putrescine	-0.20	4.73E-01	K10536
Arthrobacter	Putrescine	-0.23	4.11E-01	K10536
Psychrobacter	Putrescine	-0.31	2.56E-01	K10536
Microterricola	Putrescine	-0.34	2.11E-01	K10536
Vibrio	Putrescine	-0.48	7.35E-02	K10536
Chryseobacterium	Putrescine	-0.48	7.13E-02	K10536
Paeniglutamicibacter	Putrescine	-0.51	5.42E-02	K10536
Leucobacter	Putrescine	-0.52	4.56E-02	K10536
Lactobacillus	Putrescine	-0.53	4.11E-02	K10536
Rhodococcus	Putrescine	-0.63	1.26E-02	K10536
Galactobacter	Putrescine	-0.66	7.50E-03	K10536
Serratia	Putrescine	-0.69	4.57E-03	K10536
Kluyvera	Putrescine	-0.69	4.48E-03	K10536
Providencia	Putrescine	-0.70	3.78E-03	K10536
Acinetobacter	Putrescine	-0.70	3.40E-03	K10536
Shewanella	Putrescine	-0.78	5.67E-04	K10536
Myroides	Putrescine	-0.83	1.11E-04	K10536
Pseudomonas	Putrescine	-0.92	1.32E-06	K10536
Aeromonas	Putrescine	-0.96	6.79E-09	K10536
Lysobacter	Putrescine	0.98	3.31E-11	K12251
Bacteroides	Putrescine	0.36	1.86E-01	K12251
Oceanimonas	Putrescine	0.23	4.11E-01	K12251
Empedobacter	Putrescine	-0.27	3.34E-01	K12251
Psychrobacter	Putrescine	-0.30	2.82E-01	K12251
Chryseobacterium	Putrescine	-0.38	1.61E-01	K12251
Aeromonas	Putrescine	-0.65	8.39E-03	K12251
Acinetobacter	Putrescine	-0.79	5.19E-04	K12251
Halomonas	Putrescine	-0.86	3.35E-05	K12251
Pseudoalteromonas	Putrescine	-0.89	8.97E-06	K12251
Pseudomonas	Putrescine	-0.90	4.13E-06	K12251
Pseudosulfitobacter	Putrescine	0.97	5.99E-09	K01480
Pelagivirga	Putrescine	0.96	1.04E-08	K01480
Sulfitobacter	Putrescine	0.93	4.53E-07	K01480
Andreesenia	Putrescine	0.75	1.24E-03	K01480

Tissierella	Putrescine	0.71	3.16E-03	K01480
Agrococcus	Putrescine	0.66	7.93E-03	K01480
Ornithobacterium	Putrescine	0.43	1.08E-01	K01480
Brevibacterium	Putrescine	0.33	2.32E-01	K01480
Morganella	Putrescine	0.31	2.68E-01	K01480
Lentibacter	Putrescine	0.25	3.73E-01	K01480
Paracoccus	Putrescine	0.18	5.32E-01	K01480
Leucobacter	Putrescine	0.08	7.81E-01	K01480
Citrobacter	Putrescine	0.08	7.87E-01	K01480
Microbacterium	Putrescine	-0.01	9.80E-01	K01480
Kocuria	Putrescine	-0.09	7.60E-01	K01480
Corynebacterium	Putrescine	-0.12	6.62E-01	K01480
Arthrobacter	Putrescine	-0.25	3.73E-01	K01480
Macrococcus	Putrescine	-0.27	3.31E-01	K01480
Kaistella	Putrescine	-0.35	2.06E-01	K01480
Arcobacter	Putrescine	-0.37	1.76E-01	K01480
Bizionia	Putrescine	-0.39	1.48E-01	K01480
Proteus	Putrescine	-0.46	8.39E-02	K01480
Providencia	Putrescine	-0.53	4.32E-02	K01480
Lysinibacillus	Putrescine	-0.58	2.26E-02	K01480
Serratia	Putrescine	-0.59	2.19E-02	K01480
Chryseobacterium	Putrescine	-0.59	2.15E-02	K01480
Galactobacter	Putrescine	-0.62	1.36E-02	K01480
Mycobacteroides	Putrescine	-0.66	7.07E-03	K01480
Halomonas	Putrescine	-0.68	5.47E-03	K01480
Hafnia	Putrescine	-0.72	2.33E-03	K01480
Pseudomonas	Putrescine	-0.77	8.14E-04	K01480
Aequorivita	Putrescine	-0.81	2.93E-04	K01480
Staphylococcus	Putrescine	-0.81	2.85E-04	K01480
Paeniglutamicibacter	Putrescine	-0.81	2.21E-04	K01480
Myroides	Putrescine	-0.83	1.46E-04	K01480
Vibrio	Putrescine	-0.84	8.02E-05	K01480
Pseudoalteromonas	Putrescine	-0.86	4.42E-05	K01480
Shewanella	Putrescine	-0.88	1.95E-05	K01480
Pelagivirga	Putrescine	0.95	3.19E-08	K01476
Lysobacter	Putrescine	0.95	4.15E-08	K01476
Vagococcus	Putrescine	0.91	2.18E-06	K01476
Paracoccus	Putrescine	0.86	3.26E-05	K01476
Brevibacillus	Putrescine	0.84	9.34E-05	K01476
Ancrocorticia	Putrescine	0.74	1.67E-03	K01476
Microbacterium	Putrescine	0.51	5.00E-02	K01476
Pusillimonas	Putrescine	0.30	2.83E-01	K01476
Psychrobacter	Putrescine	0.20	4.80E-01	K01476
Enterococcus	Putrescine	0.17	5.33E-01	K01476
Planomicrobium	Putrescine	0.16	5.59E-01	K01476
Tissierella	Putrescine	-0.05	8.72E-01	K01476
Macrococcus	Putrescine	-0.12	6.75E-01	K01476
Roseovarius	Putrescine	-0.14	6.14E-01	K01476
Citrobacter	Putrescine	-0.18	5.23E-01	K01476
Serratia	Putrescine	-0.27	3.33E-01	K01476
Kluyvera	Putrescine	-0.28	3.07E-01	K01476

Myroides	Putrescine	-0.47	7.38E-02	K01476
Staphylococcus	Putrescine	-0.67	6.58E-03	K01476
Kurthia	Putrescine	-0.80	3.88E-04	K01476
Ruegeria	Putrescine	0.96	2.90E-08	K01581
Sedimentitalea	Putrescine	0.74	1.79E-03	K01581
Paracoccus	Putrescine	0.69	4.29E-03	K01581
Pelagivirga	Putrescine	0.69	4.43E-03	K01581
Massilibacillus	Putrescine	0.57	2.71E-02	K01581
Lelliottia	Putrescine	0.45	9.31E-02	K01581
Psychrobacter	Putrescine	0.42	1.23E-01	K01581
Enterobacter	Putrescine	0.39	1.50E-01	K01581
Morganella	Putrescine	0.34	2.10E-01	K01581
Citrobacter	Putrescine	0.23	4.19E-01	K01581
Lentibacter	Putrescine	0.00	9.87E-01	K01581
Proteus	Putrescine	-0.02	9.40E-01	K01581
Raoultella	Putrescine	-0.08	7.64E-01	K01581
Escherichia	Putrescine	-0.25	3.68E-01	K01581
Acinetobacter	Putrescine	-0.33	2.27E-01	K01581
Buttiauxella	Putrescine	-0.35	2.01E-01	K01581
Klebsiella	Putrescine	-0.47	7.38E-02	K01581
Providencia	Putrescine	-0.49	6.17E-02	K01581
Hafnia	Putrescine	-0.53	4.32E-02	K01581
Pseudocitrobacter	Putrescine	-0.55	3.42E-02	K01581
Kluyvera	Putrescine	-0.65	8.92E-03	K01581
Serratia	Putrescine	-0.69	4.48E-03	K01581
Moraxella	Putrescine	-0.79	4.13E-04	K01581
Aeromonas	Putrescine	-0.81	2.29E-04	K01581
Shewanella	Putrescine	-0.87	3.10E-05	K01581
Colwellia	Putrescine	-0.89	1.14E-05	K01581
Pseudomonas	Putrescine	-0.98	7.05E-11	K01581
Salmonella	Cadaverine	0.80	3.42E-04	K23385
Mixta	Cadaverine	0.27	3.35E-01	K23385
Citrobacter	Cadaverine	0.03	9.13E-01	K23385
Hafnia	Cadaverine	-0.61	1.64E-02	K23385
Caldisalinibacter	Cadaverine	0.76	9.54E-04	K01582
Gottschalkia	Cadaverine	0.71	3.16E-03	K01582
Anaerosphaera	Cadaverine	0.69	4.38E-03	K01582
Citrobacter	Cadaverine	0.55	3.38E-02	K01582
Enterobacter	Cadaverine	0.39	1.49E-01	K01582
Vibrio	Cadaverine	0.19	4.88E-01	K01582
Raoultella	Cadaverine	0.17	5.51E-01	K01582
Pseudocitrobacter	Cadaverine	0.11	7.00E-01	K01582
Lelliottia	Cadaverine	-0.22	4.41E-01	K01582
Aeromonas	Cadaverine	-0.50	6.02E-02	K01582
Obesumbacterium	Cadaverine	-0.52	4.53E-02	K01582
Escherichia	Cadaverine	-0.65	9.00E-03	K01582
Hafnia	Cadaverine	-0.65	8.05E-03	K01582
Serratia	Cadaverine	-0.81	2.37E-04	K01582
Morganella	Histamine	0.42	1.21E-01	K01590
Raoultella	Histamine	0.00	9.89E-01	K01590
Acinetobacter	Histamine	-0.86	3.49E-05	K01590

Aeromonas	Histamine	-0.62	1.38E-02	K01590
Vibrio	Spermidine	0.74	1.53E-03	K13746
Vibrio	Spermidine	0.79	4.12E-04	K13747
Arcobacter	Spermidine	0.65	8.09E-03	K13747
Pseudomonas	Spermidine	0.49	6.12E-02	K13747
Pseudoarcobacter	Spermidine	0.43	1.13E-01	K13747
Oceanisphaera	Spermidine	0.38	1.66E-01	K13747
Psychrobacter	Spermidine	-0.09	7.47E-01	K13747
Bacteroides	Spermidine	-0.24	3.84E-01	K13747
Paracoccus	Spermidine	-0.84	8.18E-05	K13747
Actibacterium	Spermidine	-0.84	8.18E-05	K13747
Myroides	Spermidine	0.85	5.19E-05	K00797
Lelliottia	Spermidine	0.72	2.25E-03	K00797
Serratia	Spermidine	0.62	1.41E-02	K00797
Pseudoalteromonas	Spermidine	0.53	4.37E-02	K00797
Chryseobacterium	Spermidine	0.51	5.27E-02	K00797
Shewanella	Spermidine	0.49	6.43E-02	K00797
Arcobacter	Spermidine	0.47	7.48E-02	K00797
Comamonas	Spermidine	0.46	8.18E-02	K00797
Photorhabdus	Spermidine	0.45	8.93E-02	K00797
Raoultella	Spermidine	0.42	1.19E-01	K00797
Pseudomonas	Spermidine	0.27	3.30E-01	K00797
Rhodopirellula	Spermidine	0.12	6.76E-01	K00797
Halomonas	Spermidine	0.10	7.23E-01	K00797
Macrococcus	Spermidine	0.02	9.46E-01	K00797
Stenotrophomonas	Spermidine	-0.05	8.51E-01	K00797
Lysobacter	Spermidine	-0.46	8.45E-02	K00797
Candidatus Berkiella	Spermidine	-0.55	3.25E-02	K00797
Brevibacterium	Spermidine	-0.69	4.60E-03	K00797
Psychrobacter	Spermidine	-0.76	1.13E-03	K00797
Dietzia	Spermidine	-0.88	1.46E-05	K00797

Table S5. Correlations and p-value between degradation genus and biogenic amine in *Chouguiyu.*

Genus	BAs	Correlation	*p*-Value	KO Source
Kocuria	Tryptamine	0.76	9.94E-04	K00274
Flavobacterium	Tryptamine	0.68	5.04E-03	K00274
Tessaracoccus	Tryptamine	0.63	1.26E-02	K00274
Staphylococcus	Tryptamine	0.59	2.17E-02	K00274
Rhodococcus	Tryptamine	0.59	2.17E-02	K00274
Oceanisphaera	Tryptamine	0.49	6.17E-02	K00274
Yoonia	Tryptamine	0.45	9.21E-02	K00274
Psychrobacter	Tryptamine	0.43	1.14E-01	K00274
Halomonas	Tryptamine	0.39	1.47E-01	K00274
Pseudoalteromonas	Tryptamine	0.31	2.56E-01	K00274
Roseovarius	Tryptamine	0.27	3.24E-01	K00274
Lysobacter	Tryptamine	0.23	4.06E-01	K00274
Primorskyibacter	Tryptamine	0.18	5.29E-01	K00274
Dietzia	Tryptamine	0.16	5.62E-01	K00274
Pelagivirga	Tryptamine	0.10	7.21E-01	K00274

Paracoccus	Tryptamine	0.08	7.76E-01	K00274
Pseudorhodobacter	Tryptamine	-0.04	8.90E-01	K00274
Pseudomonas	Tryptamine	-0.10	7.15E-01	K00274
Gillisia	Tryptamine	-0.12	6.69E-01	K00274
Primorskyibacter	Tyramine	0.93	3.30E-07	K00274
Lysobacter	Tyramine	0.92	9.46E-07	K00274
Roseovarius	Tyramine	0.87	2.90E-05	K00274
Dietzia	Tyramine	0.85	6.35E-05	K00274
Paracoccus	Tyramine	0.84	9.64E-05	K00274
Gillisia	Tyramine	0.83	1.16E-04	K00274
Tessaracoccus	Tyramine	0.75	1.33E-03	K00274
Pseudorhodobacter	Tyramine	0.68	4.84E-03	K00274
Pelagivirga	Tyramine	0.68	5.47E-03	K00274
Yoonia	Tyramine	0.44	1.01E-01	K00274
Halomonas	Tyramine	0.35	2.05E-01	K00274
Oceanisphaera	Tyramine	0.27	3.30E-01	K00274
Flavobacterium	Tyramine	0.13	6.52E-01	K00274
Psychrobacter	Tyramine	-0.02	9.56E-01	K00274
Kocuria	Tyramine	-0.31	2.69E-01	K00274
Staphylococcus	Tyramine	-0.57	2.71E-02	K00274
Rhodococcus	Tyramine	-0.57	2.71E-02	K00274
Pseudoalteromonas	Tyramine	-0.88	1.85E-05	K00274
Pseudomonas	Tyramine	-0.99	2.38E-14	K00274
Brevibacterium	Tyramine	0.98	5.77E-10	K00276
Agrococcus	Tyramine	0.95	3.36E-08	K00276
Paracoccus	Tyramine	0.94	1.16E-07	K00276
Psychrobacter	Tyramine	0.76	9.14E-04	K00276
Pseudocitrobacter	Tyramine	0.17	5.53E-01	K00276
Raoultella	Tyramine	-0.20	4.85E-01	K00276
Leclercia	Tyramine	-0.24	3.98E-01	K00276
Paeniglutamicibacter	Tyramine	-0.53	4.27E-02	K00276
Kluyvera	Tyramine	-0.56	3.08E-02	K00276
Hafnia	Tyramine	-0.64	1.02E-02	K00276
Kocuria	Tyramine	-0.70	3.84E-03	K00276
Galactobacter	Tyramine	-0.70	3.53E-03	K00276
Arthrobacter	Tyramine	-0.92	1.51E-06	K00276
Acinetobacter	Tyramine	-0.93	3.52E-07	K00276
Lactococcus	Putrescine	0.76	9.10E-04	K09251
Enterobacter	Putrescine	0.40	1.41E-01	K09251
Raoultella	Putrescine	0.10	7.28E-01	K09251
Kluyvera	Putrescine	-0.64	1.03E-02	K09251
Hafnia	Putrescine	-0.64	9.96E-03	K09251
Lelliottia	Putrescine	-0.70	3.73E-03	K09251
Citrobacter	Putrescine	-0.73	1.95E-03	K09251
Pseudomonas	Putrescine	-0.99	7.47E-12	K12256

Aeromonas	Putrescine	-0.93	6.07E-07	K12256
Halomonas	Putrescine	-0.62	1.27E-02	K12256
Shewanella	Putrescine	-0.61	1.47E-02	K12256
Lentibacter	Putrescine	-0.44	1.03E-01	K12256
Acinetobacter	Putrescine	-0.36	1.85E-01	K12256
Oceanimonas	Putrescine	0.16	5.59E-01	K12256
Leisingera	Putrescine	0.59	2.16E-02	K12256
Agrococcus	Putrescine	0.59	2.06E-02	K12256
Hoeflea	Putrescine	0.78	5.83E-04	K12256
Roseovarius	Putrescine	0.85	5.96E-05	K12256
Sulfitobacter	Putrescine	0.86	3.38E-05	K12256
Pelagivirga	Putrescine	0.88	1.90E-05	K12256
Pseudoalteromonas	Putrescine	-0.89	7.71E-06	K00797
Shewanella	Putrescine	-0.85	5.36E-05	K00797
Lelliottia	Putrescine	-0.80	3.05E-04	K00797
Myroides	Putrescine	-0.78	6.10E-04	K00797
Chryseobacterium	Putrescine	-0.77	7.65E-04	K00797
Comamonas	Putrescine	-0.75	1.36E-03	K00797
Photorhabdus	Putrescine	-0.59	2.19E-02	K00797
Serratia	Putrescine	-0.57	2.56E-02	K00797
Macrococcus	Putrescine	-0.41	1.30E-01	K00797
Halomonas	Putrescine	-0.40	1.39E-01	K00797
Raoultella	Putrescine	-0.31	2.54E-01	K00797
Arcobacter	Putrescine	-0.13	6.33E-01	K00797
Stenotrophomonas	Putrescine	0.04	8.91E-01	K00797
Rhodopirellula	Putrescine	0.10	7.36E-01	K00797
Pseudomonas	Putrescine	0.17	5.55E-01	K00797
Candidatus Berkiella	Putrescine	0.19	4.96E-01	K00797
Psychrobacter	Putrescine	0.50	5.73E-02	K00797
Dietzia	Putrescine	0.61	1.49E-02	K00797
Brevibacterium	Putrescine	0.82	2.04E-04	K00797
Lysobacter	Putrescine	0.82	1.86E-04	K00797
Vibrio	Putrescine	-0.85	5.12E-05	K13746
Enterobacter	Putrescine	-0.89	7.00E-06	K09470
Serratia	Putrescine	-0.71	2.95E-03	K09470
Acinetobacter	Putrescine	-0.70	3.80E-03	K09470
Hafnia	Putrescine	-0.45	8.99E-02	K09470
Klebsiella	Putrescine	-0.40	1.41E-01	K09470
Pseudocitrobacter	Putrescine	0.15	5.94E-01	K09470
Raoultella	Putrescine	0.32	2.39E-01	K09470
Citrobacter	Putrescine	0.36	1.93E-01	K09470
Oceanisphaera	Putrescine	0.58	2.36E-02	K09470
Vibrio	Putrescine	-0.95	5.86E-08	K00657
Aeromonas	Putrescine	-0.89	8.22E-06	K00657
Pseudoalteromonas	Putrescine	-0.84	8.47E-05	K00657

Carnobacterium	Putrescine	-0.80	2.95E-04	K00657
Kluyvera	Putrescine	-0.76	1.08E-03	K00657
Myroides	Putrescine	-0.73	2.00E-03	K00657
Hafnia	Putrescine	-0.72	2.41E-03	K00657
Serratia	Putrescine	-0.65	9.31E-03	K00657
Weissella	Putrescine	-0.65	9.39E-03	K00657
Mammaliicoccus	Putrescine	-0.60	1.86E-02	K00657
Shewanella	Putrescine	-0.55	3.35E-02	K00657
Proteus	Putrescine	-0.50	5.68E-02	K00657
Staphylococcus	Putrescine	-0.25	3.61E-01	K00657
Halomonas	Putrescine	-0.19	4.96E-01	K00657
Providencia	Putrescine	0.03	9.06E-01	K00657
Lactococcus	Putrescine	0.07	8.00E-01	K00657
Marinilactibacillus	Putrescine	0.11	6.88E-01	K00657
Moellerella	Putrescine	0.16	5.59E-01	K00657
Leucobacter	Putrescine	0.16	5.59E-01	K00657
Morganella	Putrescine	0.30	2.79E-01	K00657
Bacteroides	Putrescine	0.37	1.73E-01	K00657
Alkalibacterium	Putrescine	0.38	1.60E-01	K00657
Eubacterium	Putrescine	0.52	4.95E-02	K00657
Kosakonia	Putrescine	0.56	2.84E-02	K00657
Citrobacter	Putrescine	0.57	2.54E-02	K00657
Aequorivita	Putrescine	0.71	3.14E-03	K00657
Vagococcus	Putrescine	0.72	2.44E-03	K00657
Jeotgalibaca	Putrescine	0.81	2.79E-04	K00657
Salinicoccus	Putrescine	0.92	9.30E-07	K00657
Enterococcus	Putrescine	0.93	5.96E-07	K00657
Bacillus	Putrescine	0.93	3.49E-07	K00657
Oceanobacillus	Putrescine	0.94	2.09E-07	K00657
Lactococcus	Cadaverine	0.71	3.03E-03	K09251
Enterobacter	Cadaverine	0.53	4.40E-02	K09251
Raoultella	Cadaverine	0.21	4.44E-01	K09251
Kluyvera	Cadaverine	-0.51	5.45E-02	K09251
Hafnia	Cadaverine	-0.57	2.67E-02	K09251
Lelliottia	Cadaverine	-0.66	7.11E-03	K09251
Citrobacter	Cadaverine	-0.73	1.96E-03	K09251
Kocuria	Phenylethylamine	0.76	9.94E-04	K00274
Flavobacterium	Phenylethylamine	0.68	5.04E-03	K00274
Tessaracoccus	Phenylethylamine	0.63	1.26E-02	K00274
Staphylococcus	Phenylethylamine	0.59	2.17E-02	K00274
Rhodococcus	Phenylethylamine	0.59	2.17E-02	K00274
Oceanisphaera	Phenylethylamine	0.49	6.17E-02	K00274
Yoonia	Phenylethylamine	0.45	9.21E-02	K00274
Psychrobacter	Phenylethylamine	0.43	1.14E-01	K00274
Halomonas	Phenylethylamine	0.39	1.47E-01	K00274

Pseudoalteromonas	Phenylethylamine	0.31	2.56E-01	K00274
Roseovarius	Phenylethylamine	0.27	3.24E-01	K00274
Lysobacter	Phenylethylamine	0.23	4.06E-01	K00274
Primorskyibacter	Phenylethylamine	0.18	5.29E-01	K00274
Dietzia	Phenylethylamine	0.16	5.62E-01	K00274
Pelagivirga	Phenylethylamine	0.10	7.21E-01	K00274
Paracoccus	Phenylethylamine	0.08	7.76E-01	K00274
Pseudorhodobacter	Phenylethylamine	-0.04	8.90E-01	K00274
Pseudomonas	Phenylethylamine	-0.10	7.15E-01	K00274
Gillisia	Phenylethylamine	-0.12	6.69E-01	K00274
Leclercia	Phenylethylamine	0.67	6.71E-03	K00276
Paeniglutamicibacter	Phenylethylamine	0.65	8.40E-03	K00276
Galactobacter	Phenylethylamine	0.55	3.25E-02	K00276
Raoultella	Phenylethylamine	0.53	4.08E-02	K00276
Kluyvera	Phenylethylamine	0.49	6.28E-02	K00276
Hafnia	Phenylethylamine	0.46	8.12E-02	K00276
Kocuria	Phenylethylamine	0.44	9.90E-02	K00276
Pseudocitrobacter	Phenylethylamine	0.27	3.35E-01	K00276
Acinetobacter	Phenylethylamine	0.24	3.89E-01	K00276
Brevibacterium	Phenylethylamine	0.20	4.72E-01	K00276
Paracoccus	Phenylethylamine	0.05	8.53E-01	K00276
Psychrobacter	Phenylethylamine	0.05	8.72E-01	K00276
Agrococcus	Phenylethylamine	-0.10	7.11E-01	K00276
Arthrobacter	Phenylethylamine	-0.22	4.41E-01	K00276
